# Prevalence and Mortality of Cardiovascular-Kidney-Metabolic Syndrome in China: A Nationwide Population-Based Study

**DOI:** 10.1016/j.jacasi.2025.04.007

**Published:** 2025-06-10

**Authors:** Congyi Zheng, Anping Cai, Muyi Sun, Xin Wang, Qingjie Song, Xuyan Pei, Xue Cao, Yixin Tian, Gregory Y.H. Lip, Gianfranco Parati, Zengwu Wang, Yingqing Feng, Zhen Zhou

**Affiliations:** a Division of Prevention and Community Health, National Center for Cardiovascular Disease, Fuwai Hospital, Peking Union Medical College and Chinese Academy of Medical Sciences, Beijing, China; b Department of Cardiology, Hypertension Research Laboratory, Guangdong Cardiovascular Institute, Guangdong Provincial People’s Hospital, Guangdong Academy of Medical Sciences, Southern Medical University, Guangzhou, Guangdong Province, China; c School of Public Health, Bengbu Medical University, Bengbu, Anhui Province, China; d Department of Epidemiology, School of Public Health, Medical College of Soochow University, Suzhou, Jiangsu Province, China; e Liverpool Centre for Cardiovascular Science at University of Liverpool, Liverpool John Moores University and Liverpool Heart and Chest Hospital, Liverpool, United Kingdom; f Department of Clinical Medicine, Aalborg University, Aalborg, Denmark; g Medical University of Bialystok, Bialystok, Poland; h Department of Cardiology, IRCCS, Ospedale San Luca, Istituto Auxologico Italiano, Milan, Italy; i Department of Medicine and Surgery, University of Milano-Bicocca, Milan, Italy; j School of Public Health and Preventive Medicine, Monash University, Melbourne, Victoria, Australia

**Keywords:** cardiovascular disease, chronic kidney disease, metabolic syndrome, mortality, prevalence

## Abstract

**Background:**

Cardiovascular-kidney-metabolic (CKM) syndrome is a novel staging framework used to evaluate CKM health. The burden of CKM syndrome in China is relatively unknown, and such data may inform future health priority.

**Objectives:**

The purpose of this study was to assess the prevalence and mortality risk across CKM stages.

**Methods:**

Nationally representative populations (n = 33,685) were included from the China Hypertension Survey. The weighted prevalence of each CKM stage was calculated. All-cause, cardiovascular (CV), and non-CV death associated with CKM stages were analyzed using Cox regression analysis. Population attributable fraction (PAF) was calculated to estimate the mortality burden related to each CKM stage.

**Results:**

Between 2012 and 2015, 18.8% of Chinese adults met criteria for stage 0, 15.5% for stage 1, 42.1% for stage 2, 14.7% for stage 3, and 8.9% for stage 4, with advanced stage (stages 3-4) was 23.6%. After 5-year follow-up, compared with stage 0, adjusted HR for all-cause death in stage 1 was 0.77 (95% CI: 0.51-1.15), stage 2 was 1.36 (95% CI: 1.04-1.77), stage 3 was 2.47 (95% CI: 1.91-3.19), and stage 4 was 4.00 (95% CI: 3.07-5.22). Similarly, adjusted HRs for CV death and non-CV death progressively increased from stage 2 to 4 (both *P*-trend values < 0.001). For all-cause, CV, and non-CV death, PAFs increased with advancing CKM stages. For instance, for all-cause death, PAFs caused by stages 2, 3, and 4 were 13.4%, 18.6%, and 22.0%.

**Conclusions:**

Poor CKM health is widespread in China, underscoring the urgent need for collaborative and comprehensive management strategies to tackle CKM syndrome epidemic.

Cardiovascular disease (CVD), kidney disease, and metabolic disorders such as obesity and type 2 diabetes frequently coexist and are pathophysiologically inter-related.^1^^,^^2^ Concurrence of these conditions significantly increases mortality risk.^2^^,^^3^ In light of growing burdens and to promote multidisciplinary and inclusive care, the American Heart Association has introduced a novel staging framework known as cardiovascular-kidney-metabolic (CKM) syndrome.^4^

Recently, a Chinese study involving 1,110 health care workers found that nearly 90% of male participants and 60% of female participants met the criteria for CKM syndrome (stage 1 or higher).^5^ Another 2 cohort studies also found high prevalence of CKM syndrome in Chinese adults.^6^^,^^7^ The Kailuan Study, which included 97,777 community adults in northern China, showed that risk of all-cause mortality increased with advancing CKM stages.^8^ These findings provide preliminary data into the burden of CKM syndrome in Chinese adults. However, these studies are either subject to small sample size and single-center study,^5^ lack of imaging data to define subclinical CVD or laboratory data to identify albuminuria,^6^^,^^7^ or predominantly male participants and lack of cause-specific mortality data.^8^ Considering that nearly 330 million adults in China are living with CVD,^9^ 85 million have obesity,^10^ 130 million are diagnosed with type 2 diabetes,^11^ and 120 million have chronic kidney disease (CKD),^12^ a more comprehensive analysis using nationally representative data is highly needed to accurately assess the burden of CKM syndrome in the general Chinese population.

Therefore, leveraging data from the China Hypertension Survey (2012-2015),^13^ a nationally representative study, we sought to assess the overall prevalence of CKM syndrome, as well as the specific prevalence by sociodemographic factors, in the general Chinese population. We further investigated the cause-specific mortality of CKM syndrome. These findings would help enhance our knowledge of the scope and prognostic impact of CKM syndrome in China and may inform future health priority.

## Methods

### Study design and participants

This retrospective cohort study utilized data from the China Hypertension Survey (2012-2015), which employed a stratified, multistage, and random sampling method to obtain nationally representative subjects from overall 31 provinces in mainland China.^14^ For the study of CKM syndrome, we included subjects who participated in the echocardiography substudy. Briefly, during the survey, individuals aged 35 years or older from 14 provinces were randomly selected for the echocardiography substudy, and 34,994 (response rate, 62.5%) natives or inhabitants who lived in the investigation points for more than 6 months were recruited. Those with missing data on the variables required for CKM syndrome classification were excluded (n = 1,309), leaving a total of 33,685 subjects included in this study. Written informed consent was obtained from all participants. The Ethics Committee of Fuwai Hospital (Beijing, China) approved this study. All performances were conducted according to the Declaration of Helsinki.

### Study variables and data collection

A standardized questionnaire developed by the coordinating center, Fuwai Hospital (Beijing, China), was used by trained staff to collect information on sociodemographic factors (age, sex, urbanity, and education attainment), health behavior (smoking and drinking status), and family history of CVD. Height was measured to the nearest 0.5 cm using a fixed measurement tape and a standard right-angle device with participant barefoot. Body weight was measured using an OMRON body fat and weight measurement device (V-body HBF-371, OMRON) with participants wearing light clothing. Body mass index (BMI) was calculated as weight in kilograms divided by height in square meters. Waist circumference was measured to the nearest 0.1 cm using a cloth tape placed directly on the participant’s skin at the midpoint between the lower edge of the last rib and the iliac crest along the midaxillary line. Blood pressure (BP) was measured 3 times on the right arm positioned at heart level using the OMRON HBP-1300 professional portable BP monitor (OMRON). Measurements were taken after the participant had been sitting at rest for 5 minutes, with a 30-second interval between each measurement. The average of the 3 BP readings was recorded. Laboratory analyses were performed in a central core laboratory (Beijing Adicon Clinical Laboratories, Inc, Beijing, China) using standardized techniques, and all blood samples were collected in the morning after at least 8 hours of overnight fasting. Serum glucose was measured using an enzymatic method, and serum total cholesterol, high-density lipoprotein cholesterol, and triglycerides were measured using enzymatic methods with an autoanalyzer. Echocardiographic examinations were performed by trained sonographers following a standardized protocol.

### Definitions of CKM syndrome stages and its components

According to the American Heart Association Scientific Statement,^4^ CKM syndrome is divided into stages 0-4: stage 0 represents no CKM risk factors, stage 1 is defined by excess or dysfunctional adiposity, stage 2 is defined by metabolic risk factors and CKD, stage 3 is defined by subclinical CVD in CKM, and stage 4 is defined by clinical CVD in CKM. Stages 3 and 4 were combined as advanced CKM stage.^15^ Definitions of the components of CKM syndrome are presented in Supplemental Table 1. In this study, overweight and obesity was defined by BMI ≥24 and ≥28 kg/m^2^, respectively.^16^ Hypertension was defined by systolic BP *≥*140 mm Hg, diastolic BP ≥90 mm Hg, or use of antihypertensive medications in the last 2 weeks,^13^ and hypertriglyceridemia was defined by triglyceride ≥1.5 mmol/L. Metabolic syndrome was defined by the presence of ≥3 of the following metabolic factors: 1) waist circumference ≥90 cm for men and ≥85 cm for women; 2) high-density lipoprotein cholesterol <1.0 mmol/L for men and <1.3 mmol/L for women; 3) triglycerides ≥1.7 mmol/L; 4) elevated BP (systolic BP *≥*130 mm Hg and/or diastolic BP ≥80 mm Hg and/or use of antihypertensive medications); and 5) fasting blood glucose ≥5.5 mmol/L. Diabetes mellitus was defined by fasting plasma glucose level ≥7.0 mmol/L or receiving antidiabetic medications. CKD was defined by decreased estimated glomerular filtration rate (eGFR) (<60 mL/min/1.73 m^2^) or presence of albuminuria (urinary albumin to creatinine ratio [UACR] ≥30 mg/g) based on the Kidney Disease Improving Global Outcomes guideline (KDIGO).^17^ Subclinical heart failure was defined by presence of enlarged left atrium, enlarged left ventricle, left ventricular hypertrophy, or left ventricular diastolic dysfunction on echocardiogram.^18^ Grades 4 and 5 CKD was defined by eGFR 15 to 29 and <15 mL/min/1.73 m^2^, respectively. Very high risk per KDIGO classification was defined as follows: 1) UACR ≥300 mg/g and eGFR ≤45 to 59 mL/min/1.73 m^2^; 2) UACR ≥30 mg/g and eGFR ≤30 to 44 mL/min/1.73 m^2^; or 3) eGFR ≤29 mL/min/1.73 m^2^. High predicted 10-year CVD risk was calculated using the American Heart Association’s PREVENT (Predict Risk of cardiovascular disease EVENTs) Equations.^19^ Coronary heart disease was identified based on self-reported history of myocardial infarction, percutaneous coronary intervention, or coronary artery bypass grafting. Heart failure was identified based on self-reported history or presence of heart failure symptom, stroke was identified based on self-reported history, peripheral arterial disease was identified based on ankle-brachial index <0.9, and atrial fibrillation was identified based on self-reported history or electrocardiogram findings.

### Study outcomes

The study outcomes included all-cause, cardiovascular (CV), and non-CV death. Specifically, if the cause of death was primarily attributed to underlying CVD, such as coronary heart disease, stroke, or heart failure, it was defined as CV death; and if the cause of death was related to non-CVD conditions, such as cancer or respiratory disease, it was classified as non-CV death. The events were retrospectively collected by study staff through direct contact with the participants’ immediate relatives and health care providers. These data were further verified using China’s National Mortality Surveillance System for death registration and mortality surveillance. The accuracy of these data has been validated.^20^

### Statistical analysis

Continuous variables with a normal distribution were presented as mean ± SD, otherwise were presented as median (IQR); and categorical variables were presented as frequency (percentage). The prevalence of each CKM stage in the general Chinese population was estimated using data from the overall participants (n = 33,685), along with stratified prevalence rates based on age, sex, urbanity, and education attainment. To enhance the representativeness of the survey sample, adjustments were made for differential selection probabilities and the complex sampling design. Survey weights were calculated based on the 2010 China population census data and the sampling scheme.^21^ These weights accounted for factors such as oversampling of specific age subgroups, nonresponse, and demographic differences between the sample and the general population. To address the multistage stratified sampling design, rates were calculated using the PROC SURVEYFREQ procedure, and differences across the age, sex, urbanity, and education attainment subgroups were tested using PROC SURVEYLOGISTIC in SAS.^13^ Weighted prevalence were summarized as mean and 95% CI. The prevalence of advanced CKM stage (stages 3-4) was also compared among the sociodemographic subgroups. Among participants with follow-up data on vital status (n = 23,683), 5-year cumulative rates of all-cause, CV, and non-CV death were displayed using Kaplan-Meier curve and compared by log-rank test. Cox proportional hazards regression analysis was conducted to evaluate the association between CKM stages and risks of all-cause, CV, and non-CV death, with stage 0 served as referenced group. HRs and 95% CI: were calculated. The regression models were adjusted for age, sex, urbanity, education attainment, smoking and drinking status, and family history of CVD. The population attributable fraction (PAF) is an epidemiologic measure widely used to assess the public health impact of exposures in populations,^22^ and to estimate the mortality burden related to each CKM stage in the general Chinese population, we calculated PAF using the equation: PAF = P×(HR − 1)/(P×[HR − 1] + 1), where P represents the population prevalence of the specific CKM stage, and HR refers to the adjusted HR for mortality related to that stage. The 95% CI: for PAF were derived through 1,000 times bootstrapping. The number of attributable deaths for each CKM stage was calculated by multiplying the PAF by the estimated number of all-cause (n = 9,119,739), CV (n = 4,105,957), and non-CV (n = 5,013,782) deaths in China in 2018 based on the data from the China Health Statistical Report of 2018. The event number in stage 1 was extremely low and no PAF was calculated. All statistical analyses were conducted using survey modules of SAS software version 9.4 (SAS Institute, Inc). Two-sided *P* values <0.05 were considered statistically significant.

## Results

### Characteristics of participants across CKM stages

The mean age of the cohort was 57.1 years, 57.8% were women, 50.6% were from rural areas, and 78.9% had education attainment below high school (Supplemental Table 2). The percentage of participants with individual component of CKM syndrome was as follows (Table 1): overweight/obesity (56.3%), abdominal obesity (39.6%), hypertriglycemia (33.8%), hypertension (43.9%), metabolic syndrome (19.3%), diabetes mellitus (11.3%), CKD (23.9%), subclinical CVD (25.1%), and clinical CVD (9.6%). The percentage of participants with stages 0 to 4 was 16.9%, 13.7%, 39.3%, 20.5%, and 9.6%, respectively. There were somewhat differences in baseline characteristics between participants with and without follow-up. Specifically, individuals lost to follow-up were more likely to have hypertension and diabetes mellitus but less likely to have metabolic syndrome and subclinical CVD. In addition, they were less likely to have advanced CKM stage (Supplemental Table 2).

**Table 1:** Baseline Characteristics Across CKM Stages

	Stage 0 (n = 5,688)	Stage 1 (n = 4,623)	Stage 2 (n = 13,233)	Stage 3 (n = 6,908)	Stage 4 (n = 3,233)
Age, y	51.5 ± 11.3	50.0 ± 10.6	54.8 ± 11.0	68.5 ± 11.9	64.5 ± 12.6
Age group					
35-44 y	2,220 (39.0)	1,747 (37.7)	2,897 (21.9)	334 (4.8)	293 (9.1)
45-64 y	2,635 (46.3)	2,288 (49.5)	7,340 (55.5)	1,851 (26.8)	1,154 (35.7)
≥65 y	833 (14.6)	588 (12.7)	2,996 (22.6)	4,723 (68.4)	1,786 (55.2)
Sex					
Men	2,366 (41.6)	1,873 (40.5)	5,917 (44.7)	3,509 (50.8)	1,569 (48.5)
Women	3,322 (58.4)	2,750 (59.5)	7,316 (55.3)	3,399 (49.2)	1,664 (51.5)
Urbanity					
Urban	2,671 (47.0)	2,385 (51.6)	6,768 (51.1)	3,123 (45.2)	1,680 (52.0)
Rural area	3,017 (53.0)	2,238 (48.4)	6,465 (48.9)	3,785 (54.8)	1,553 (48.0)
Education					
<High school	4,240 (74.7)	3,442 (74.5)	10,135 (76.8)	5,999 (87.0)	2,720 (84.3)
≥High school	1,437 (25.3)	1,176 (25.5)	3,068 (23.2)	895 (13.0)	507 (15.7)
Current smoker	1,463 (25.7)	933 (20.2)	3,106 (23.5)	1,781 (25.8)	693 (21.4)
Current drinker	1,452 (25.7)	1,282 (27.8)	3,828 (29.0)	1,754 (25.5)	836 (25.9)
Family history of CVD	456 (8.0)	502 (10.9)	2,290 (17.3)	1,142 (16.5)	729 (24.1)
SBP, mm Hg	117.0 ± 11.0	120.1 ± 10.1	136.2 ± 18.3	145.3 ± 22.3	141.1 ± 22.4
DBP, mm Hg	71.2 ± 7.9	74.7 ± 9.7	76.8 ± 11.1	75.8 ± 11.6	76.9 ± 12.0
Heart rate, beats/min	75.0 ± 10.5	74.8 ± 9.7	76.8 ± 11.2	75.3 ± 11.4	76.9 ± 11.9
Waist circumference in men, cm	77.2 ± 6.3	89.0 ± 7.1	88.3 ± 9.3	85.7 ± 10.7	88.7 ± 10.0
Waist circumference in women, cm	73.4 ± 5.7	85.2 ± 7.5	84.7 ± 9.8	84.5 ± 11.0	86.2 ± 10.6
Body mass index, kg/m^2^	21.4 ± 1.7	26.2 ± 2.4	25.4 ± 3.4	24.7 ± 4.0	25.4 ± 3.7
Total cholesterol, mmol/L	4.5 ± 0.9	4.6 ± 0.9	4.9 ± 1.0	4.9 ± 1.0	4.8 ± 1.0
LDL cholesterol, mmol/L	2.7 ± 0.7	2.8 ± 0.8	2.9 ± 0.9	2.9 ± 0.8	2.8 ± 0.9
HDL cholesterol, mmol/L	1.5 ± 0.3	1.4 ± 0.3	1.3 ± 0.3	1.4 ± 0.4	1.3 ± 0.3
Triglyceride, mmol/L	0.84 (0.39)	0.95 (0.42)	1.56 (1.11)	1.19 (0.92)	1.26 (0.94)
Fasting plasma glucose, mmol/L	5.0 ± 0.6	5.1 ± 0.6	5.7 ± 1.7	5.9 ± 1.8	6.1 ± 1.9
Overweight/obesity	0	4,291 (93.0)	8,503 (65.2)	3,820 (56.1)	2,073 (65.8)
Abdominal obesity	0	2,281 (49.5)	6,284 (48.3)	2,929 (43.0)	1,636 (51.5)
Hypertriglyceridemia	0	0	6,525 (54.4)	2,248 (35.3)	1,074 (37.9)
Hypertension	0	0	7,631 (58.2)	4,863 (70.8)	2,179 (67.7)
Metabolic syndrome	0	0	3,992 (30.1)	1,679 (24.3)	853 (26.4)
Diabetes mellitus	0	0	1,555 (12.7)	1,338 (20.0)	604 (20.4)
Chronic kidney disease	0	0	3,156 (29.0)	2,351 (39.4)	972 (36.3)
Subclinical CVD	0	0	0	6,908 (100.0)	1,570 (48.6)
Subclinical HF	0	0	0	4,235 (64.0)	976 (31.9)
Risk equivalents of subclinical CVD	0	0	0	154 (2.3)	43 (1.5)
High predicted 10-y CVD risk in men	0	0	0	2,247 (64.0)	579 (36.9)
High predicted 10-y CVD risk in women	0	0	0	1,539 (45.3)	398 (24.0)
Clinical CVD	0	0	0	0	3,233 (100.0)
CHD	0	0	0	0	429 (13.3)
HF	0	0	0	0	362 (11.2)
Stroke	0	0	0	0	870 (26.9)
PAD	0	0	0	0	1,525 (48.1)
Atrial fibrillation	0	0	0	0	384 (11.9)
Medication	0	0	3,845 (29.0)	3,033 (43.9)	1,635 (50.6)
Antihypertensive	0	0	3,155 (23.8)	2,708 (39.2)	1,456 (45.0)
Lipid-lowering	0	0	644 (4.9)	323 (4.7)	362 (11.2)
Antidiabetic	0	0	752 (5.7)	715 (10.4)	387 (12.0)

Values are mean ± SD or n (%).

CHD = coronary heart disease; CKM = cardiovascular-kidney-metabolic; CVD = cardiovascular disease; DBP = diastolic blood pressure; HDL = high-density lipoprotein; HF = heart failure; LDL = low-density lipoprotein; PAD = peripheral arterial disease; SBP = systolic blood pressure.

### Weighted prevalence of CKM stages

Between 2012 and 2015, 18.8% (95% CI: 18.2%-19.4%) of Chinese adults met criteria for stage 0, 15.5% (95% CI: 15.0%-16.1%) for stage 1, 42.1% (95% CI: 41.3%-42.8%) for stage 2, 14.7% (95% CI: 14.2%-15.2%) for stage 3, and 8.9% (95% CI: 8.4%-9.3%) for stage 4, with advanced stage was 23.6% (95% CI: 22.9%-24.2%) (Table 2). Only 31.5% (95% CI: 25.7%-37.3%) of adults aged 35 to 44 years had stage 0. Among subjects aged 35 to 44 and 45 to 64 years, stage 2 was most prevalent, while stage 3 was most prevalent in those aged 65 years or older. The prevalence of advanced stage increased with age, reaching 56.6% among individuals aged ≥65 years, which was ≈7 times and ≈2.5 times higher than those aged 35 to 44 years and 45 to 64 years, respectively. Within the sex, urbanity, and education attainment subgroups, stage 2 was most prevalent, affecting approximately 2 in 5 of Chinese adults. More than 1 in 5 had advanced stage in both sexes, with men having a 2.6% higher prevalence than women. Rural areas had a significantly higher prevalence of advanced stage than urban areas, with a difference of 5.6%. Individuals with education attainment below high school had a 12.6% higher prevalence of advanced stage compared with those with education attainment at or above high school.

**Table 2:** Weighted Prevalence of CKM Stages

	Prevalence, % (95% CI)						Advanced Stage	
	Stage 0 (n = 5,688)	Stage 1 (n = 4,623)	Stage 2 (n = 13,233)	Stage 3 (n = 6,908)	Stage 4 (n = 3,233)	Advanced stage (n = 10,141)	PR (95% CI)	PR^a^ (95% CI)
Total	18.8 (18.2-19.4)	15.5 (15.0-16.1)	42.1 (41.3-42.8)	14.7 (14.2-15.2)	8.9 (8.4-9.3)	23.6 (22.9-24.2)	—	—
Age group, y								
35-44	31.5 (25.7-37.3)	24.0 (19.1-29.8)	36.5 (30.2-42.7)	2.0 (0.9-4.4)	6.0 (3.1-11.3)	8.0 (4.7-13.3)	Ref	Ref
45-64	16.5 (15.8-17.3)	14.6 (13.9-15.3)	47.6 (46.5-48.6)	12.6 (11.9-13.3)	8.7 (8.1-9.4)	21.3 (20.4-22.2)	2.68 (2.45-2.93)	2.60 (2.38-2.85)
≥65	8.4 (7.7-9.2)	5.8 (5.2-6.4)	29.1 (29.0-30.3)	41.5 (40.2-42.7)	15.1 (14.3-16.1)	56.6 (55.4-57.9)	16.13 (14.74-17.66)	15.52 (14.16-17.00)
Sex								
Men	17.0 (16.2-18.2)	14.0 (13.2-14.8)	44.1 (43.0-45.2)	15.5 (14.8-16.2)	9.4 (8.7-10.2)	24.9 (24.0-25.9)	Ref	Ref
Women	20.5 (19.7-21.4)	16.9 (16.2-17.7)	40.2 (39.2-41.2)	14.0 (13.4-14.7)	8.3 (7.7-8.9)	22.3 (21.5-23.2)	0.76 (0.72-0.79)	0.76 (0.72-0.80)
Urbanity								
Urban	17.8 (17.0-18.7)	16.8 (16.0-17.7)	45.0 (43.9-46.1)	12.9 (12.3-13.6)	7.5 (7.0-8.0)	20.4 (19.6-21.2)	Ref	Ref
Rural area	19.6 (18.8-20.4)	14.5 (13.8-15.3)	39.8 (38.8-40.9)	16.1 (15.4-16.8)	9.9 (9.2-10.7)	26.0 (25.1-26.9)	1.21 (1.07-1.18)	1.21 (1.15-1.28)
Education								
≥High school	21.9 (20.5-23.3)	17.6 (163-18.9)	46.0 (44.3-47.7)	8.6 (7.8-9.4)	6.0 (5.2-6.9)	14.6 (13.5-15.8)	Ref	Ref
<High school	18.1 (17.4-18.7)	15.0 (14.4-15.6)	40.1 (40.2-41.8)	16.3 (15.8-16.9)	9.6 (9.1-10.2)	26.0 (25.2-26.7)	1.98 (1.86-2.11)	1.33 (1.23-1.43)

Advanced stage indicated stages 3 and 4.

CKM = cardiovascular-kidney-metabolic; PR = prevalence ratio.

a Adjusted for age, sex, urbanity, and education.

### All-cause and cause-specific mortality across CKM stages

**Figure 1:**
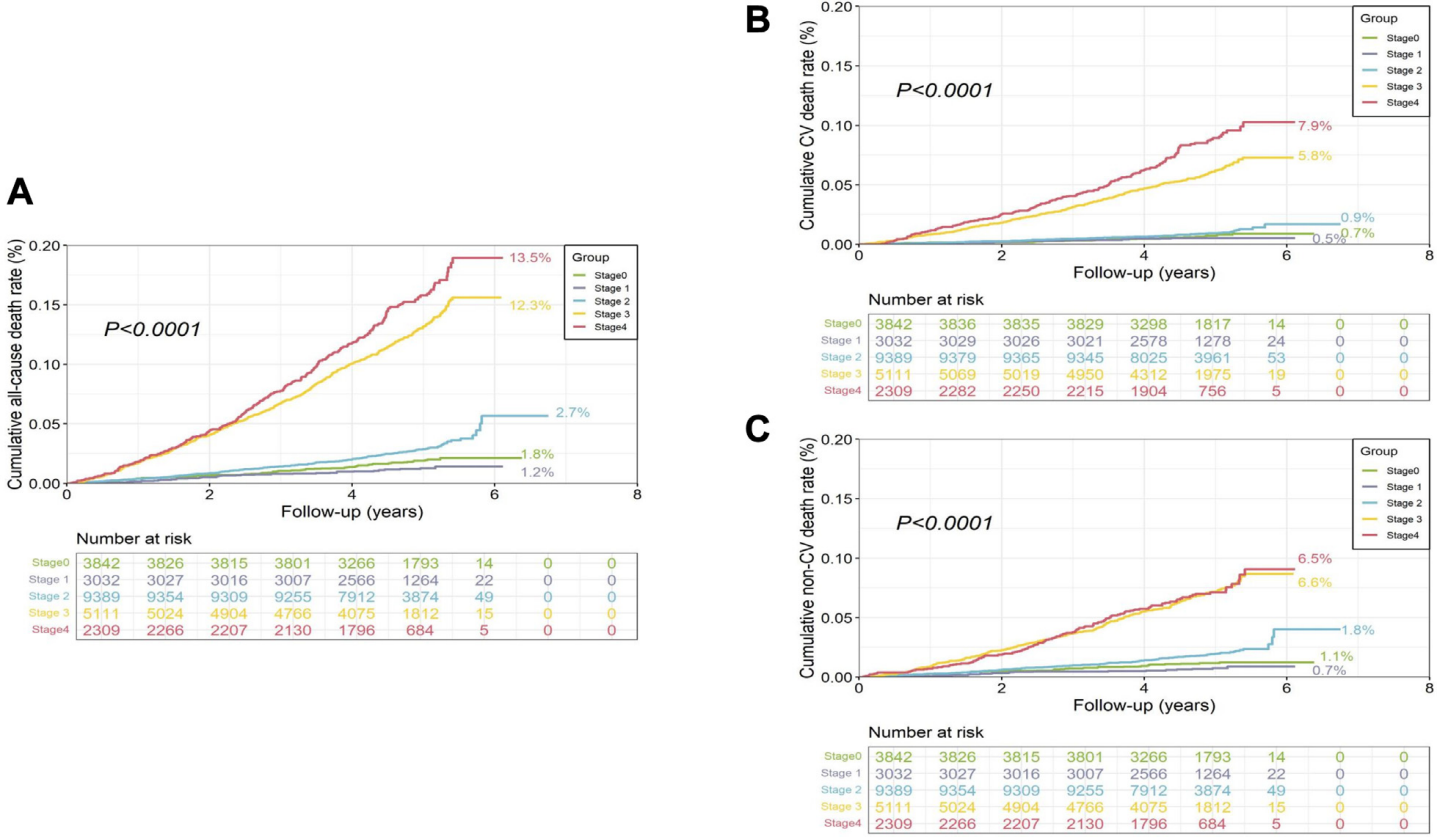
Cumulative Rate of Mortality at 5-Year Follow-Up 5-year cumulative rate of all-cause death (A), cardiovascular (CV) death (B), and non-CV death (C) were significantly different across cardiovascular-kidney-metabolic (CKM) stages.

From stages 0 to 4, the 5-year cumulative rates of all-cause death were 1.5%, 1.0%, 2.3%, 10.0%, and 11.6% (log-rank *P <* 0.0001) (Figure 1A); CV death rates were 0.6%, 0.4%, 0.8%, 4.7%, and 6.4% (log-rank *P <* 0.0001) (Figure 1B); and non-CV death rates were 0.9%, 0.6%, 1.5%, 5.3%, and 5.2% (log-rank *P <* 0.0001) (Figure 1C). Compared with stage 0, adjusted HR for all-cause death in stage 1 was 0.77 (95% CI: 0.51-1.15), stage 2 was 1.36 (95% CI: 1.04-1.77), stage 3 was 2.47 (95% CI: 1.91-3.19), and stage 4 was 4.00 (95% CI: 3.07-5.22), with *P*-trend value < 0.001 (Table 3). Similarly, compared with stage 0, adjusted HRs for CV death and non-CV death significantly increased from stages 2 to 4 (both *P*-trend values < 0.001).

**Table 3:** Mortality Risk Across CKM Stages

	Stage 0 (n = 3,842)	Stage 1 (n = 3,032)	Stage 2 (n = 9,389)	Stage 3 (n = 5,111)	Stage 4 (n = 2,309)	*P* Trend Value
All-cause death						
Person-y	18,052.4	14,181.5	43,704.0	22,745.9	9,972.3	
Number of events	69	36	258	631	331	
IR (95% CI) per 1,000 person-y	3.8 (3.0-4.8)	2.5 (1.8-3.5)	5.9 (5.2-6.7)	27.7 (25.6-30.0)	33.2 (30.0-36.9)	<0.001
Unadjusted HR (95% CI)	Ref	0.67 (0.45-1.00)	1.55 (1.19-2.02)	7.37 (5.75-9.45)	8.93 (6.89-11.58)	<0.001
Adjusted HR (95% CI)^a^	Ref	0.77 (0.51-1.15)	1.36 (1.04-1.77)	2.47 (1.91-3.19)	4.00 (3.07-5.22)	<0.001
Cardiovascular death						
Person-y	18,163.1	14,232.0	44,059.5	23,453.2	10,292.5	
Number of events	26	15	86	296	182	
IR (95% CI) per 1,000 person-y	1.4 (1.0-2.1)	1.1 (0.6-1.7)	2.0 (1.6-2.4)	12.6 (11.2-14.1)	17.7 (15.3-20.4)	<0.001
Unadjusted HR (95% CI)	Ref	0.74 (0.39-1.40)	1.37 (0.88-2.12)	8.91 (5.96-13.30)	12.65 (8.39-19.09)	<0.001
Adjusted HR (95% CI)	Ref	0.87 (0.46-1.65)	1.16 (0.75-1.81)	2.52 (1.67-3.80)	4.87 (3.20-7.41)	<0.001
Noncardiovascular death						
Person-y	18,052.4	14,181.5	43,704.0	22,745.9	9,972.3	
Number of events	43	21	172	335	149	
IR (95% CI) per 1,000 person-y	2.4 (1.8-3.2)	1.5 (0.9-2.2)	3.9 (3.3-4.6)	14.7 (13.2-16.4)	14.9 (12.7-17.5)	<0.001
Unadjusted HR (95% CI)	Ref	0.62 (0.37-1.05)	1.66 (1.19-2.32)	6.26 (4.56-8.60)	6.42 (4.57-9.01)	<0.001
Adjusted HR (95% CI)	Ref	0.71 (0.42-1.19)	1.47 (1.05-2.07)	2.39 (1.72-3.32)	3.19 (2.25-4.52)	<0.001

CKM = cardiovascular kidney-metabolic; IR = incidence rate.

a Adjusted for age, sex, urbanity, education, smoking, drinking, and family history of CVD.

### Estimated mortality cases attributable to each CKM stage

For all-cause, CV, and non-CV death, PAFs increased from stage 2 to stage 4 (Table 4). For example, for all-cause death, PAFs caused by stages 2, 3, and 4 were 13.4% (95% CI: 13.2%-13.6%), 18.6% (95% CI: 18.0%-19.2%), and 22.0% (95% CI: 21.0%-23.0%), respectively. It was estimated that 1,221,113 (95% CI: 1,199,246-1,243,020) deaths were attributable to stage 2, 1,694,448 (95% CI: 1,642,465-1,748,254) deaths were attributable to stage 3, and 2,009,079 (95% CI: 1,916,969-2,101,188) deaths were attributable to stage 4 in China in 2018. The PAF caused by stage 2 was lower for CV death compared with non-CV death (6.4% [95% CI: 6.3%-6.6%] vs 16.8% [95% CI: 16.5%-17.1%]), while the PAF caused by stage 4 was higher for CV death compared with non-CV death (24.6% [95% CI: 23.7%-25.6%] vs 17.1% [95% CI: 16.3%-17.9%]). Estimated CV and non-CV deaths attributable to each CKM stage are presented in Table 4.

**Table 4:** Estimated Attributable Risk and Number of Mortality Events Attributable to CKM Stages

CKM stage	PAF, % (95% CI)	Cases Attributable to CKM in the Present Cohort (95% CI)	Cases Attributable to CKM in China (95% CI)
All-cause death			
Stage 1	/	/	/
Stage 2	13.4 (13.2-13.6)	177 (174-181)	1,221,113 (1,199,246-1,243,020)
Stage 3	18.6 (18.0-19.2)	246 (239-254)	1,694,448 (1,642,465-1,748,254)
Stage 4	22.0 (21.0-23.0)	292 (279-305)	2,009,079 (1,916,969-2,101,188)
Cardiovascular death			
Stage 1	/	/	/
Stage 2	6.4 (6.3-6.6)	39 (38-40)	264,013 (259,086-268,940)
Stage 3	17.2 (16.7-17.8)	115 (112-119)	705,403 (684,874-731,271)
Stage 4	24.6 (23.7-25.6)	162 (155-169)	1,096,701 (1,049,483-1,143,920)
Noncardiovascular death			
Stage 1	/	/	/
Stage 2	16.8 (16.5-17.1)	125 (123-127)	841,814 (827,775-856,354)
Stage 3	17.8 (17.2-18.3)	134 (130-138)	889,946 (862,371-918,525)
Stage 4	17.1 (16.3-17.9)	129 (123-135)	857,357 (815,742-898,971)

CKM = cardiovascular-kidney-metabolic; PAF = population attributable fraction.

## Discussion

To our knowledge, this is the first nationally representative study to assess the prevalence and mortality risk of CKM syndrome in the general Chinese population (Central Illustration). We found that nearly 80% of Chinese adults met criteria for CKM syndrome (stage 1 or higher), and 23.6% met for advanced stage. Significant sociodemographic disparities in advanced stage prevalence were noted, with older age, male sex, rural residence, and lower educational attainment associated with higher risk. Importantly, the risks of all-cause, CV, and non-CV death, as well as the PAFs associated with each CKM stage, progressively increased from stages 2 to 4. These findings highlight the urgent need for collaborative and comprehensive management strategies to tackle CKM syndrome epidemic in China.

**Central Illustration:**
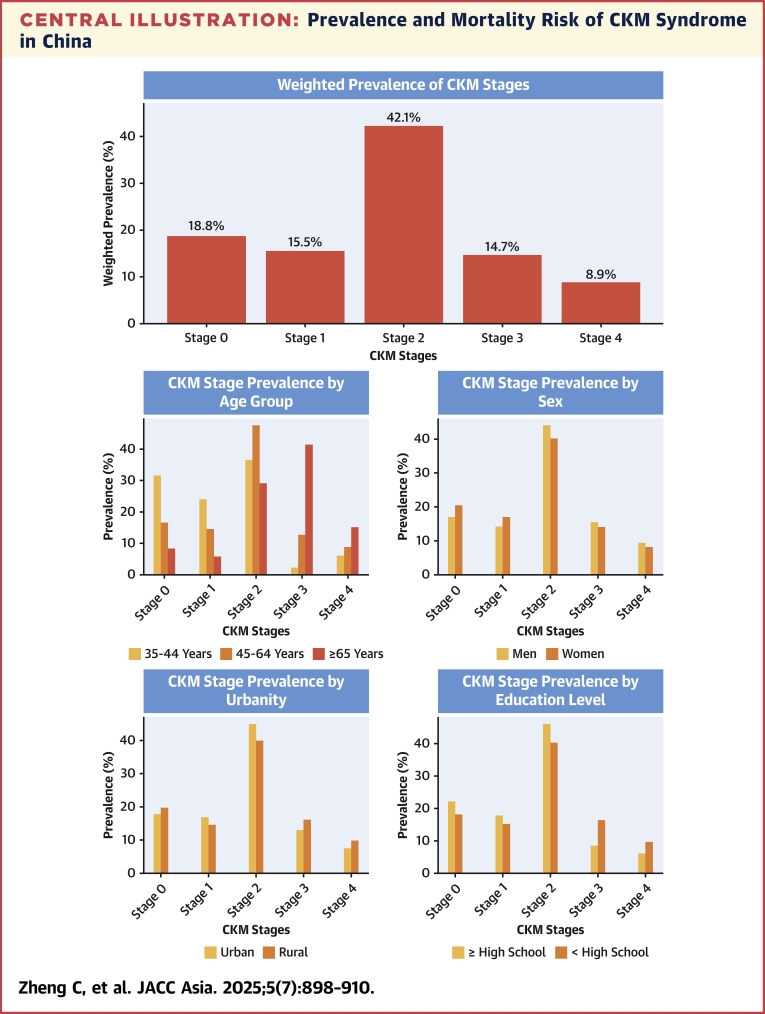

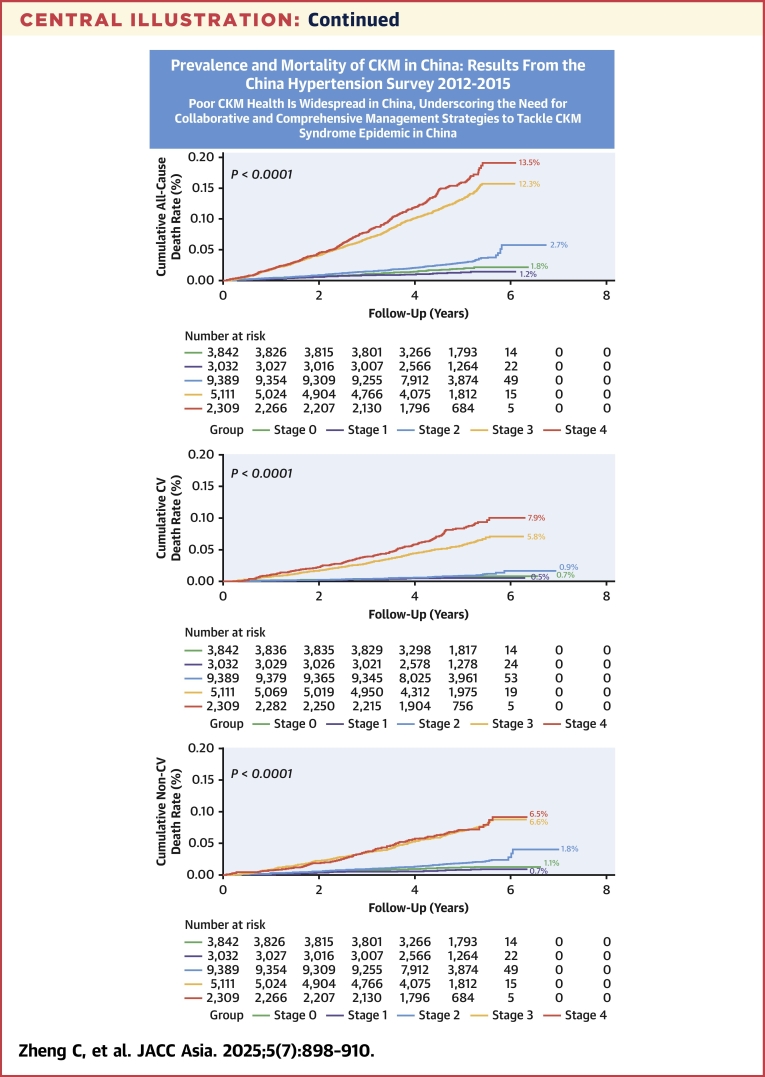
Prevalence and Mortality Risk of CKM Syndrome in China Between 2012 and 2015, the advanced cardiovascular-kidney-metabolic (CKM) stage in Chinese adults aged ≥35 years or older was up to 23.6% (95% CI: 22.9%-24.2%). The 5-year’s cumulative rate of all-cause death, cardiovascular (CV) death, and non-CV death increased from stage 2 to stage 4.

### Prevalence of CKM syndrome

Since its introduction in 2023,^4^ few studies have reported the nationwide prevalence of CKM syndrome. Aggarwal et al,^15^ using data from the National Health and Nutrition Examination Survey (NHANES) (2011-2020), found that 10.6% of U.S. adults met criteria for stage 0, 25.9% for stage 1, 49.0% for stage 2, 5.4% for stage 3, and 9.2% for stage 4, with advanced stage occurring in 14.6%.^15^ Similar to the NHANES findings, we also found that stage 2 was most prevalent, affecting 42.1% of Chinese adults. However, the prevalence of advanced stage was higher in the Chinese population compared with the U.S. population, primarily driven by the greater prevalence of stage 3 (14.7% vs 5.4%). Differences in the criteria used might partially explain these variations. In this study, echocardiography was used to define subclinical heart failure, which was not available in the NHANES study.^15^ Although it is important to note that neither this study nor the NHANES studies collected data on serum cardiac biomarkers and coronary computed tomography angiography. If such data had been available, the prevalence of stage 3 might have been even higher. Both our study and the U.S. study showed that the prevalence of stage 0 was <20%,^15^ demonstrating the widespread nature of poor CKM health globally.

In addition to assessing the overall prevalence of CKM syndrome, its prevalence across several sociodemographic subgroups has also been examined in this and prior studies.^6^^,^^7^^,^^15^^,^^23^ Aggarwal et al^15^ reported that individuals aged ≥65 years (vs 20-44 and 45-64 years) were more likely to have advanced stage. This finding aligns with our study and 2 other studies conducted in Chinese adults.^6^^,^^7^ Furthermore, both ours and NHANES studies showed that only 18% to 30% of subjects <45 years of age had stage 0.^15^^,^^23^ These findings underscore the urgent need of implementing early prevention and management strategies to address CKM syndrome epidemic across all age groups including young adults. The higher prevalence of advanced CKM stage in men (vs women) suggests a need for sex-specific approaches to prevention and intervention. Furthermore, additional attention will be needed to address urbanity and education-based differences in the assessment and management across CKM stages. This is particularly crucial considering the disparities in health care resources and health literacy between urban and rural populations, as well as between individuals with different levels of education attainment in China.^24^, ^25^, ^26^ In line with this study, prior studies of U.S. adults also showed that those with lower socioeconomic status such as lower education attainment were at a higher risk of having advanced CKM stage.^27^^,^^28^ Tailored strategies to overcome these barriers will be essential to ensure equitable access to effective prevention, diagnosis, and treatment,^29^ ultimately reducing the burden of CKM syndrome in underserved populations. Furthermore, considering aging population and rapid urbanization in China over the past decades, the weighted prevalence of CKM among the elderly and urban populations may have changed significantly. Future research should incorporate updated census data or alternative weighting methods to evaluate whether these demographic shifts significantly impact the estimated CKM burden, particularly in older adults and urban residents.

### Prognostic impact of CKM syndrome

The mortality burden associated with individual component of CKM syndrome has been well studied. For example, high systolic BP accounted for 19.2% of all-cause deaths worldwide in 2019.^30^ Type 2 diabetes contributed to 1.4 million deaths globally in 2019.^31^ Deaths related to CKD have risen significantly, with the global mortality rate increasing by 41.5% from 1990 to 2017.^32^ Since recent introduction,^4^ there has been increasing interest in understanding the prognostic impact of CKM syndrome. Findings from the Kailuan study provide timely and valuable insights into this area. Compared with stage 0, the adjusted risk of all-cause death increased progressively, from 1.24-fold in stage 1 to 3.73-fold in stage 4.^8^ Although our study showed a stepwise increase in all-cause death risk from stages 2 to 4, and individuals with stage 1 exhibited a nonsignificant reduction in adjusted mortality risk. The reasons for these findings are unclear, but it could be caused by the possibility that individuals at stage 1 (vs those at stage 0) were younger and less likely to be current smokers. In addition, they were more likely to live in urban areas, where health care resources were more abundant compared with rural areas.^33^ These favorable characteristics might mitigate the adverse effects of excess and/or dysfunctional adiposity. In addition, although BMI and waist circumference are easily measured, both have limitations. Specifically, BMI cannot differentiate between lean mass and fat mass, and waist circumference cannot distinguish between subcutaneous fat and visceral fat, each of which exerts distinct biological effects on CV and metabolic system.^34^ Further research may be needed to investigate whether measures of fat mass (vs BMI) and visceral fat (vs waist circumference) could more accurately reflect mortality risk in individuals classified as having stage 1 CKM syndrome.

Extending prior study,^8^ we further assessed the impact of CKM syndrome on CV and non-CV death. These analyses could help us to better understand how CKM syndrome contributes to death from CV and non-CV conditions, thus informing more targeted preventive and therapeutic strategies. Specifically, as indicated by the PFAs, stage 2 of CKM, characterized by presence of metabolic risk factors or CKD, had greater contribution to non-CV death than to CV death (PAF: 16.8% vs 6.4%). These findings emphasize the need for inclusive and multidisciplinary approach to risk assessment and management, focusing not only on CV health but also on metabolic and renal conditions.^4^ In contrast, stage 4 of CKM, featured by presence of clinical CVD, contributed more to CV death than non-CV death (PAF: 24.6% vs 17.1%), suggesting the need for intensive CV management, close monitoring for complications, and a targeted approach to reducing mortality from CV causes.^4^

Understanding the mortality burden of CKM syndrome is crucial for clinical efforts, especially in light of the growing number of therapeutic management options available for these conditions. For example, results of 2 post hoc analyses of heart failure clinical trials showed that both sacubitril/valsartan and dapagliflozin safely improved clinical outcomes in patients with mildly reduced or preserved ejection fraction, and these benefits were consistent in those with and without CV-renal-metabolic conditions.^35^^,^^36^ A greater benefit was noted among those with the highest CV-renal-metabolic overlap.^36^ Semaglutide significantly reduced body weight and the incidence of CV death in obese populations with existing CVD.^37^ Furthermore, in patients with CKD and type 2 diabetes, semaglutide reduced the risk of clinically important kidney outcomes and death from CV causes.^38^ In light of CKM syndrome epidemic, further studies are urgently needed to evaluate whether the clinical benefits observed in the trials can be (if yes, and how to) be effectively translate to the general population in the real-world situations.

### Study limitations

First, data such as serum cardiac biomarkers and coronary computed tomography angiography were unavailable which might underestimate the prevalence of stage 3. Second, history of CVD was based on self-report which might result in misclassification. Third, a proportion of participants were lost to follow-up or had missing data on vital status, which could introduce selection bias. Because participants without follow-up were less likely to have advanced CKM stages, this may have led to an underestimation of CKM-related mortality risk. Fourth, because of the inherent limitation of observational design, residual confounding cannot be completely ruled out. Finally, this study used the 2010 census data for weighting, which might not fully capture recent demographic changes. Future studies should consider incorporating contemporary census data to more accurately estimate the CKM burden in China.

## Conclusions

The burden of CKM syndrome is high in China, especially among the elderly, men, people living in rural areas, and individuals with low education attainment. Multidisciplinary and inclusive care, as well as targeted screening and prevention efforts for individuals in the early stages of CKM syndrome, are urgently needed to prevent disease progression and reduce the associated mortality burden.

## Funding Support and Author Disclosures

This work was supported by the Noncommunicable Chronic Diseases-National Science and Technology Major Project of China (Grant #No. 2023ZD0508906); Noncommunicable Chronic Diseases-National Science and Technology Major Project of China (Grant #No. 2024ZD0526803); The Climbing Plan of Guangdong Provincial People's Hospital (DFJH2020022); Guangdong special funds for science and technology innovation strategy, China (Stability support for scientific research institutions affiliated to Guangdong Province-GDCI 2024); Pillar Program during the Twelfth Five-year Plan Period (No.: 2011BAI11B01); Noncommunicable Chronic Diseases-National Science and Technology Major Project (No. 2023ZD0503500); and the Major science and technology special plan project of Yunnan Province (202302AA310045). The authors have reported that they have no relationships relevant to the contents of this paper to disclose.
