# Supplementary material for: Prevalence and Mortality of Cardiovascular-Kidney-Metabolic Syndrome in China: A Nationwide Population-Based Study
Source: JACC Asia. 2025 Jun 10;5(7):898–910. doi: 10.1016/j.jacasi.2025.04.007 (PMC12277190; doi:10.1016/j.jacasi.2025.04.007)
Supplement: Supplemental Tables 1 and 2 [file mmc1.docx]

**Supplemental Table 1 Definitions of CKM stages and its components**

| **Stage** | **AHA Scientific Statement Definition** | **Modified definition in this study** |
| --- | --- | --- |
| Stage 0: No CKM health risk factors | Individuals without overweight/obesity, metabolic risk factors (hypertension, hypertriglyceridemia, MetS, diabetes), CKD, or subclinical/clinical CVD | - Overweight/obesity (BMI ≥ 24/28 kg/m^2^) - Hypertension: systolic BP ≥ 140 mm Hg, diastolic BP ≥ 90 mm Hg, or use of antihypertensive medications in the last 2 weeks - Hypertriglyceridemia: triglyceride ≥ 1.5 mmol/L - Metabolic syndrome: based on the presence of ≥ 3 of the following metabolic factors: (1) waist circumference **≥** 90 cm for men and ≥ 85 cm for women; (2) high-density lipoprotein cholesterol < 1.0 mmol/L for men and < 1.3 mmol/L for women; (3) triglycerides ≥ 1.7 mmol/L; (4) elevated BP (systolic BP ≥ 130 mm Hg and/or diastolic BP ≥ 80 mm Hg and/or use of antihypertensive medications); and (5) fasting blood glucose ≥ 5.5 mmol/L. - Diabetes: fasting plasma glucose level ≥ 7.0 mmol/L or receiving antidiabetic medications - CKD: defined as either decreased estimated glomerular filtration rate (eGFR < 60 mL/min/1.73m2) or presence of albuminuria (urinary albumin to creatinine ratio [UACR] ≥ 30 mg/g) based on the Kidney Disease Improving Global Outcomes guideline |
| Stage 1: Excess  and/or dysfunctional  adiposity | Individuals with overweight/obesity, abdominal  obesity, or dysfunctional adipose tissue, without the  presence of other metabolic risk factors or CKD   - BMI ≥25 kg/m^2^ (or ≥23 kg/m^2^ if Asian ancestry) - Waist circumference ≥ 88/102 cm in women/   men (or if Asian ancestry, ≥ 80/90 cm in women/  men) and/or   - Fasting blood glucose ≥ 100-124 mg/dL or   HbA1c between 5.7% and 6.4% |  |
| Stage 2: Metabolic  risk factors and  CKD | Individuals with metabolic risk factors  (hypertriglyceridemia (≥135 mg/dL), hypertension,  MetS, diabetes) or CKD |  |
| Stage 3: Subclinical  CVD in CKM | Subclinical ASCVD or subclinical HF among  individuals with excess/dysfunctional adiposity, other  metabolic risk factors, or CKD   - Subclinical ASCVD to be principally diagnosed   by coronary artery calcification (subclinical  atherosclerosis by coronary catheterization/CT  angiography also meets criteria)   - Subclinical HF diagnosed by elevated cardiac   biomarkers (NT-proBNP ≥ 125 pg/mL, high-sensitivity troponin T ≥ 14 ng/L for women and ≥ 22 ng/L for men, high-sensitivity troponin  I ≥ 10 ng/L for women and ≥ 12 ng/L for men)  or by echocardiographic parameters, with  combination indicating highest HF risk.  Risk equivalents of subclinical CVD   - Very high-risk CKD (G4 or G5 CKD or very high risk per KDIGO classification) - High predicted 10-y CVD risk | - Subclinical HF defined based on the echocardiographic parameter:   Enlarged LA: LA anteroposterior diameter > 4.0 cm for men and > 3.8 cm for women, or LA diameter/height > 2.61 cm/m for both sexes  Enlarged LV: LV end diastolic diameter/body surface area (BSA) > 30 mm/m2 for men and > 31 mm/m2 for women  LVH: left ventricular mass/BSA > 109 g/m^2^ for men and > 105 g/m^2^ for women  LVDD: moderate or grade II (pseudo-normal LV filling) and severe or grade III (restrictive filling) of LVDD   - G4 CKD: eGFR 15-29 ml/min/1.73m^2^ - G5 CKD: eGFR < 15ml/min/1.73m^2^ - Very high risk per KDIGO classification: UACR ≥ 300 mg/g and eGFR ≤ 45-59 ml/min/1.73m^2^; UACR ≥ 30 mg/g and eGFR ≤ 30-44 ml/min/1.73m^2^; or eGFR ≤ 29 ml/min/1.73m^2^ - High predicted 10-y CVD risk: calculated based on the |
| Stage 4: Clinical  CVD in CKM | Clinical CVD (coronary heart disease, heart failure, stroke, peripheral artery disease, AFib) among individuals with excess/dysfunctional adiposity, other metabolic risk factors, or CKD   - Stage 4a: no kidney failure - Stage 4b: kidney failure present | - CHD was defined based on self-report of prior myocardial infarction, prior percutaneous coronary intervention or coronary artery bypass grafting - HF was defined based on self-report of prior history, or presence of HF symptom - Stroke was defined based on self-report - PAD was defined based on ankle-brachial index (ABI) < 0.9 - AFib was defined based on self-report or report on the electrocardiographic examination |

CKM, Cardiovascular-Kidney-Metabolic; AHA, American Heart Association; MetS, metabolic syndrome; CKD, chronic kidney disease; CVD, cardiovascular disease; BMI, body mass index; BP, blood pressure; HbA1c, glycated hemoglobin A1c; ASCVD, atherosclerotic cardiovascular disease; CT, computed tomography; NT-proBNP, N-terminal pro-B-type natriuretic peptide; CHD, coronary heart disease; HF, heart failure; PAD, peripheral arterial disease; AFib, atrial fibrillation

**Supplemental Table 2 Baseline characteristics of the overall participants, individuals with and without follow-up**

|  | **Overall**  **(n=33685)** | **With follow-up**  **(n=23683)** | **Without follow-up**  **(n=10002)** | **P-value** |
| --- | --- | --- | --- | --- |
| Age (years) | 57.1±13.3 | 56.6±13.2 | 58.3±13.5 | <0.001 |
| Age group, n (%) |  |  |  | <0.001 |
| 35-44 years | 7491 (22.2) | 5451 (23.0) | 2040 (20.4) |  |
| 45-64 years | 15268 (45.3) | 11031 (46.6) | 4327 (42.4) |  |
| ≥ 65 years | 10926 (32.5) | 7201 (30.4) | 3725 (37.2) |  |
| Sex, n (%) |  |  |  | <0.001 |
| Men | 15234 (45.2) | 11031 (46.6) | 4230 (42.0) |  |
| Women | 18451 (57.8) | 12652 (53.4) | 5799 (58.0) |  |
| Urbanity, n (%) |  |  |  | <0.001 |
| Urban | 16627 (49.4) | 10948 (46.2) | 5679 (56.8) |  |
| Rural area | 17058 (50.6) | 12735 (53.8) | 4323 (43.2) |  |
| Education, n (%) |  |  |  | <0.001 |
| < High school | 26536 (78.9) | 18843 (79.7) | 7693 (77.1) |  |
| ≥ High school | 7083 (21.1) | 4793 (20.3) | 2290 (22.9) |  |
| Current smoker, n (%) | 7976 (23.7) | 5849 (24.7) | 2127 (21.3) | <0.001 |
| Current drinker, n (%) | 9152 (27.8) | 6585 (27.9) | 2567 (25.8) | <0.001 |
| Family history of CVD, n (%) | 5619 (15.5) | 3724 (15.7) | 1445 (14.5) | <0.001 |
| SBP (mm Hg) | 133.1±20.6 | 132.9±20.4 | 133.5±21.0 | 0.12 |
| DBP (mm Hg) | 77.4±11.2 | 77.6±11.1 | 77.0±11.1 | <0.001 |
| Heart rate (bpm) | 76.0±11.0 | 76.1±11.0 | 76.0±11.1 | 0.98 |
| Waist circumference in men (cm) | 86.1±9.9 | 86.1±9.9 | 86.2±10.0 | 0.36 |
| Waist circumference in women (cm) | 82.9±10.2 | 83.0±10.1 | 82.6±10.4 | 0.06 |
| Body mass index (kg/m^2^) | 24.7±3.6 | 24.7±3.5 | 24.7±3.8 | 0.99 |
| Total cholesterol (mmol/L) | 4.8±1.0 | 4.8±1.0 | 4.9±1.0 | <0.001 |
| LDL cholesterol (mmol/L) | 2.8±0.8 | 2.8±0.8 | 2.9±0.8 | <0.001 |
| HDL cholesterol (mmol/L) | 1.4±0.3 | 1.4±0.3 | 1.4±0.4 | <0.001 |
| Triglyceride (mmol/L) | 1.2 (0.9) | 1.2 (0.9) | 1.2 (0.9) | 0.12 |
| Fasting plasma glucose (mmol/L) | 5.6±1.6 | 5.6±16 | 5.6±1.7 | 0.11 |
| Overweight/obesity, n (%) | 18687 (56.3) | 13182 (56.3) | 5505 (56.3) | 0.96 |
| Abdominal obesity, n (%) | 13130 (39.6) | 9288 (39.7) | 3842 (39.3) | 0.53 |
| Hypertriglyceridemia, n (%) | 9847 (33.8) | 7526 (33.7) | 2321 (33.9) | 0.74 |
| Hypertension, n (%) | 14673 (43.9) | 10189 (43.3) | 4844 (45.4) | <0.001 |
| Metabolic syndrome, n (%) | 6524 (19.3) | 5015 (21.1) | 1509 (15.1) | <0.001 |
| Diabetes mellitus, n (%) | 3497 (11.3) | 2486 (10.6) | 1011 (13.8) | <0.001 |
| Chronic kidney disease, n (%) | 6479 (23.9) | 4840 (23.6) | 1639 (24.5) | 0.16 |
| Subclinical CVD, n (%) | 8478 (25.1) | 6272 (26.5) | 2206 (22.1) | <0.001 |
| Subclinical HF | 5211 (17.2) | 3888 (17.4) | 1323 (16.8) | 0.22 |
| Risk equivalents of subclinical CVD | 197 (0.6) | 139 (0.6) | 58 (0.8) | 0.04 |
| High predicted 10-y CVD risk in men | 2826 (18.5) | 2512 (19.5) | 674 (16.0) | <0.001 |
| High predicted 10-y CVD risk in women | 1937 (10.5) | 1402 (11.0) | 535 (9.2) | <0.001 |
| Clinical CVD, n (%) | 3233 (9.6) | 2309 (9.7) | 924 (9.2) | 0.15 |
| CHD | 429 (1.3) | 276 (1.2) | 153 (1.5) | 0.007 |
| HF | 362 (1.1) | 243 (1.0) | 119 (1.2) | 0.18 |
| Stroke | 870 (2.6) | 589 (2.5) | 281 (2.8) | 0.09 |
| PAD | 1525 (4.7) | 1170 (5.0) | 355 (3.8) | <0.001 |
| Atrial fibrillation | 384 (1.1) | 253 (1.1) | 131 (1.3) | 0.06 |
| CKM stage, n (%) |  |  |  | <0.001 |
| Stage 0 | 5688 (16.9) | 3842 (16.2) | 1846 (18.4) |  |
| Stage 1 | 4623 (13.7) | 3032 (12.8) | 1591 (15.9) |  |
| Stage 2 | 13233 (39.3) | 9389 (39.6) | 3844 (38.4) |  |
| Stage 3 | 6908 (20.5) | 5111 (21.6) | 1797 (18.0) |  |
| Stage 4 | 3233 (9.6) | 2309 (9.7) | 924 (9.2) |  |
| Medication, n (%) |  |  |  |  |
| Anti-hypertensive | 7319 (21.7) | 5028 (21.2) | 2291 (22.9) | <0.001 |
| Lipid-lowering | 1329 (3.9) | 922 (3.8) | 407 (4.1) | 0.44 |
| Anti-diabetic | 1854 (5.5) | 1258 (5.3) | 596 (6.0) | 0.02 |

CKM, Cardiovascular-Kidney-Metabolic; CVD, cardiovascular disease; SBP, systolic blood pressure; DBP, diastolic blood pressure; LDL, low-density lipoprotein; HDL, high-density lipoprotein; HF, heart failure, CHD, coronary heart disease; PAD, peripheral arterial disease
